# Quality of recovery in patients administered remimazolam versus those administered an inhalant agent for the maintenance of general anesthesia: a randomized control trial

**DOI:** 10.1186/s12871-022-01770-x

**Published:** 2022-07-16

**Authors:** Seung Woo Song, Yo Nam Jang, Min-Woo Yoon, Yeong-Gwan Jeon

**Affiliations:** 1 Department of Anesthesiology and Pain Medicine, Wonju College of Medicine, Yonsei University, Ilsan-ro 20, Wonju-si, Gangwon-do 26426 Republic of Korea; 2 Department of Anesthesiology and Pain Medicine, Wonju Severance Christian Hospital, Wonju-si, Republic of Korea

**Keywords:** Remimazolam, General Anesthesia, Anesthesia Recovery Period, Postoperative Nausea and Vomiting

## Abstract

**Background:**

Remimazolam is a novel intravenous benzodiazepine that is appropriate for the maintenance of anesthesia. Quality of recovery is an important component of health care quality, but there is no published randomized control trial focused on the quality of recovery in patients undergoing total intravenous anesthesia with remimazolam.

**Methods:**

This parallel-group, single-blind randomized control trial at a tertiary care medical center in South Korea was conducted to determine the difference in the quality of recovery between the patients administered remimazolam and those administered an inhalant anesthetic agent. A total of 168 patients aged 19–65 years who underwent elective laparoscopic cholecystectomy or robotic gynecologic surgery were considered for enrollment. Randomization was performed using sealed envelopes containing computer-generated random allocation sequences.

Remimazolam was administered for the maintenance of anesthesia in the remimazolam group (Group R), and desflurane was administered in the desflurane group (Group D). The induction protocol and the target value of the bispectral index were identical in both groups. Patients were blinded to the drug that was administered until they finished the postoperative questionnaire.

The main outcome measure was the decrement of the QoR-40 score on postoperative day 1 compared to the QoR-40 score on the day before surgery.

**Results:**

A total of 165 patients were analyzed. The preoperative and postoperative global QoR-40 scores were 183 and 152 (IQR 173–192 and 136–169), respectively. The perioperative decrement of the global QoR-40 score was 29.96 ± 22.49. The decrement of the QoR-40 score was smaller in Group R than in Group D (26.99 versus 32.90, respectively; mean difference 5.91, 95% confidence interval -0.96–12.79). After adjustment for sex, the type of surgery and surgical time, the administration of remimazolam resulted in a 7.03-point (95% CI 0.35–13.72) less decrement of the QoR-40 score than desflurane. There were no severe adverse events in either group.

**Conclusion:**

Total intravenous anesthesia maintained with remimazolam provides a better quality of recovery than anesthesia maintained with an inhalant agent in patients undergoing laparoscopic surgery. Additionally, postoperative nausea and vomiting were largely reduced with remimazolam.

**Trial registration:**

KCT0006288, Clinical Research Information Service (CRIS), Republic of Korea

Registration date: 23/06/2021

## Background

The quality of recovery has recently been increasingly emphasized, as the safety of anesthesia and surgery has improved significantly [1]. Quality of recovery is also associated with patient satisfaction, which is an important element of health care quality [2, 3]. The QoR-40 has been used as a measure of the quality of recovery in various studies [4–6]. This questionnaire consists of 40 items addressing five dimensions of health, and its validity and reliability have been extensively studied [4, 7].

Total intravenous anesthesia (TIVA) promotes a better quality of recovery than conventional balanced anesthesia maintained by inhaled anesthetic agents and opioids. As a hypnotic drug allowing TIVA, propofol is widely used because of its rapid onset and recovery compared to other intravenous hypnotics. Remimazolam, a novel ultrashort-acting benzodiazepine, can be utilized as an alternative drug to propofol.

To the best of the authors’ knowledge, no published randomized control trial has focused on the quality of recovery in patients undergoing TIVA with remimazolam. This study compared the quality of recovery in patients undergoing TIVA with remimazolam versus balanced anesthesia, which is currently the most common form of general anesthesia [8]. We hypothesized that TIVA conducted with remimazolam would result in a better quality of recovery than balanced anesthesia.

## Method

### Study setting

This randomized parallel-group controlled trial was approved by the Institutional Review Board of Wonju Severance Christian Hospital (CR321039; approval date: 10/06/2021) and registered with the Clinical Research Information Service of Korea (KCT0006288; registration date: 23/06/2021). The study was performed at a tertiary care university medical center in Wonju, South Korea. This study complied with the consolidated standards of reporting trials (CONSORT) guidelines.

### Participants

All consecutive patients undergoing elective laparoscopic cholecystectomy or robotic gynecologic surgery were considered for enrollment using the following inclusion criteria: patients aged 19 to 65 years old; patients with an American Society of Anesthesiologist physical status classification (ASA PS classification) of I to III; and patients with an estimated length of anesthesia of 2 hours or less. The exclusion criteria were as follows: patients undergoing ambulatory surgery; patients with a body mass index (BMI) of 30 or higher; patients who were pregnant or breastfeeding; patients with a hypersensitivity to benzodiazepine; patients planned in advance to be transferred to the intensive care unit postoperatively; patients with a history of acute narrow-angle glaucoma; patients with Child-Turcotte-Pugh class C hepatic dysfunction; patients who were unable to communicate; patients with cognitive disorders; and patients who were unable to understand the written information about the trial or the informed consent form.

### Randomization

Patients were randomly assigned (1:1) to either the remimazolam group (Group R) or the desflurane group (Group D). A random allocation sequence was created by one of the authors (SWS) using R statistical software 4.1.2 (R Core Team, Vienna, Austria). Cards indicating the group allocation were contained in sequentially numbered, opaque envelopes. One of the authors (YGJ) kept the opaque envelopes containing the group allocation until the day of the surgery on which the envelopes were opened by one of the authors (YNJ), who informed an attending anesthesiologist of the group allocation just before the induction of anesthesia.

### Study protocol

All participants received written information about the study the day before surgery. Sufficient time was allowed for the patients to learn about and understand the study before signing the informed consent form. Enrollment was mainly conducted by one of the authors (YNJ) while under the supervision of another author (YGJ). After providing written informed consent, the patients responded manually to the printed Korean questionnaire of the QoR-40, which was previously translated and evaluated by Lee et al. [1]

No premedication for anesthesia was prescribed to the patients. A dose of 2 mg of propofol and 0.8 mg of rocuronium per ideal body weight (IBW) kilogram were administered for the induction of anesthesia. After loss of consciousness, 1-2 mg*kg^-1^*hr^-1^ of remimazolam was administered to Group R and 0.7-0.9 MAC of desflurane was administered to Group D. Remifentanil was infused at a rate of 0.05–0.2 μg*kg^-1^*min^-1^ in both groups during induction and maintenance of anesthesia. The replacement of desflurane by sevoflurane in group D was allowed if the patients had reversible airflow obstruction, tachycardia possibly due to desflurane, or emergency situations that were equivalent to these.

The depth of anesthesia was monitored by the bispectral index (BIS Complete Monitoring system, Coviden Ireland Limited, Dublin, Ireland). The target BIS value was 55. The anesthesia depth was adjusted when the BIS was lower than 53 or higher than 57.

Additional rocuronium (0.15 mg per IBW kilogram) was administered upon request by the surgeon or due to the marked reduction of lung compliance. A dose of 0.3 mg of ramosetron was administered for prophylaxis of postoperative nausea and vomiting (PONV). Fentanyl (1 μg/kg) was administered on skin closure, and the infusion of remifentanil was terminated 1 minute after the administration of fentanyl. If the patient had requested patient-controlled analgesia (PCA), 10 mcg per kg of fentanyl, 0.3 mg of ramosetron and 60 mg of nefopam were mixed in 100 mL of normal saline. The basal infusion rate of the PCA device was 2 mL/hr, and the bolus was 1 mL with a minimal interval of 15 minutes.

The length of stay in the PACU was at least 30 minutes due to the protocol of the medical center, and pain was measured every 10 minutes in the PACU. Fentanyl 50 μg was administered as a rescue analgesic drug. The patients were blinded to the drug that was administered to maintain anesthesia until completion of the postoperative questionnaire. Postoperative QoR-40 was conducted 24 hours after the surgery by the patient responding to a printed form by hand.

### Variables and assessments

The primary outcome was the perioperative decrement of the QoR-40 score, which was the postoperative QoR-40 score subtracted from the preoperative QoR-40 score. The secondary outcomes were PONV, the occurrence of intraoperative hypotension, the time to extubation, the time to discharge to the PACU, postoperative pain in the PACU, the administration of rescue analgesics, and the recovery duration. The time to extubation and time to discharge to the PACU were measured from the end of the surgical procedure.

### Statistical analysis

R statistical software (version 4.1.2) was used for statistical analysis. Categorical data were analyzed by the chi-square test. The normality of continuous variables was tested by the Shapiro–Wilk test. The primary outcome and continuous variables with a normal distribution were analyzed by the t test, and other parameters were analyzed by the Wilcoxon rank sum test. A p value less than 0.05 was considered to be statistically significant.

Multivariable linear regression analysis was performed to determine the adjusted effect of the intervention. The covariates included sex and surgical time, which are known to affect the quality of recovery [9–11]. The quality of recovery can vary depending on the characteristics of the surgery itself. Therefore, the type of surgery was also analyzed as a covariate.

### Sample size

We assumed that the variability of the primary outcome would be similar to that of a previous study comparing the QoR-40 scores of patients receiving TIVA and balanced anesthesia [6]. We assumed that there would be more than a 10-point difference in the perioperative decrement of the QoR-40 score.

The alpha value was set to 0.05, and the beta value was set to 0.2. The enrollment of 76 participants to each group would be sufficient. The projected drop-out was assumed to be 10%. Therefore, 84 patients were enrolled in each group.

## Results

**Fig. 1:**
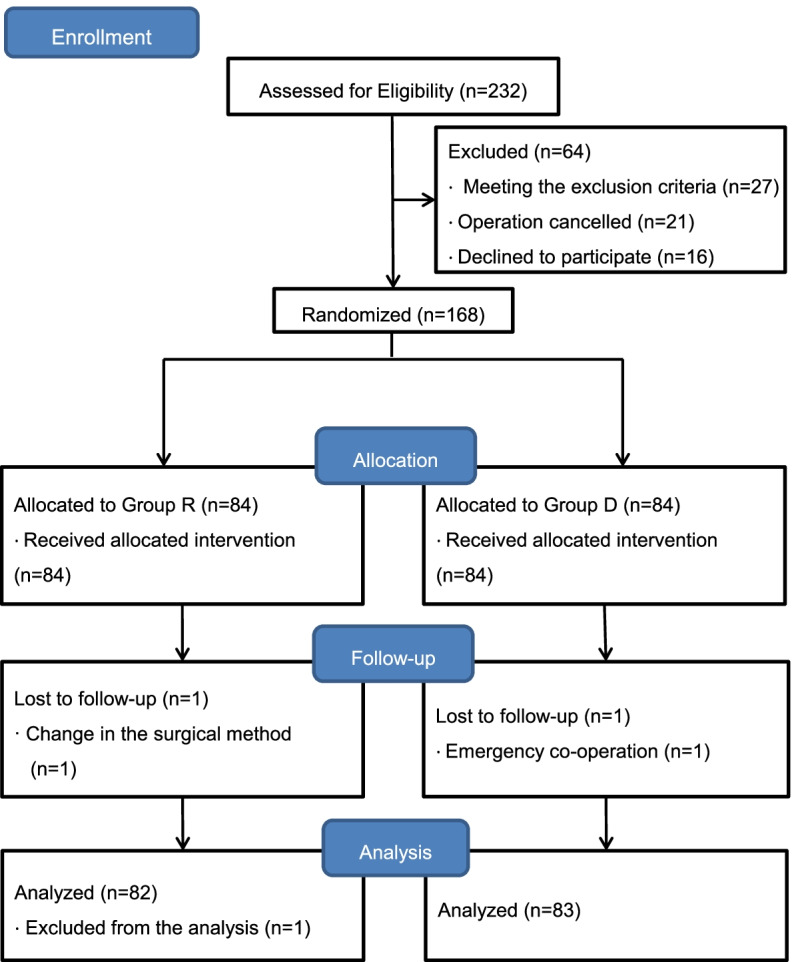
CONSORT flow diagram of the study

From June 2021 to March 2022, 232 patients were assessed for eligibility, and a total of 168 patients were enrolled (Fig. 1). One patient in Group D, who underwent laparoscopic cholecystectomy, was withdrawn due to an unexpected major gynecologic co-operation. One patient in Group R was withdrawn due to a change in the surgical method. Another patient in Group R was excluded from the analysis because the patient responded to the preoperative questionnaire using ranges rather than points.

Baseline patient characteristics are presented in Table 1. Sevoflurane was administered for three patients in Group D. The reasons for replacement for each patient were the failure of the vaporizer, tachycardia of 130 beats per minute, and the daily use of a beta-2 agonist inhaler. The injected amount of remimazolam in group R was 102.7 ± 64.4 mg. Flumazenil was administered for 16 patients (19.5%) in Group R, including 5 patients in the operating room and 11 patients in the PACU.

**Table 1:** Baseline patient characteristics

	Group R (*n*=82)	Group D (*n*=83)
Age, y (SD)	43.4 (10.4)	43.2 (9.0)
Male, n (%)	20 (24.4)	18 (21.7)
ASA PS classification, n		
I or II	77	77
III	5	6
Type of surgery, n		
Laparoscopic cholecystectomy	29	39
Robotic gynecologic surgery	53	44
Duration of surgery, min	61 (42–95)	60 (41–80)
Duration of anesthesia, min	105 (80–133.8)	100 (82.5–120)
Total dose of rocuronium, mg	50 (40–50)	45 (40–50)
Application of PCA, n (%)	42 (51.2)	38 (45.7)
Reversal of neuromuscular block by sugammadex, n (%)	63 (76.8)	53 (63.8)

*ASA PS* American Society of Anesthesiologist Physical Status, *PCA* patient-controlled analgesia, *SD* standard deviation. Continuous data that were not normally distributed are presented as the median (IQR)

The global and subdimensional QoR-40 scores were not normally distributed. The median and interquartile range of the preoperative and postoperative global QoR-40 scores of all participants were 183 (173-192) and 152 (136-169), respectively. The perioperative decrement of the global QoR-40 score was 29.96 (22.49).

The perioperative decrement of the QoR-40 score was comparable in every dimension in Group R than in Group D except for the subdimension of ‘emotional state', where remimazolam was favorable (Table 2). The decrement of the global QoR-40 score was significantly smaller in Group R than in Group D after adjustment for surgical time, type of surgery and sex (Table 3). The F-statistic, adjusted R^2^ and P value of the linear regression model were 5.46, 0.10 and < 0.001, respectively.

**Table 2:** Comparison of QoR-40 by group

Dimensions (Total points)	Group R			Group D			Difference of decrement (95% CI)
	Preop	Post-op	Decrement	Preop	Post-op	Decrement	
Emotional state (45) *	39 (36–42)	36.5 (33.25–40)	2.56 (5.12)	40 (38–42)	35 (31–39)	4.47 (5.55)	1.91 (0.27–3.55)
Physical comfort (60)	57 (53.25–58)	48 (43–53)	7.63 (7.47)	56 (52–58)	46 (41.5–50.5)	9.12 (7.29)	1.49 (-0.78–3.76)
Psychological support (35)	32 (28.25–35)	28 (24.25–32)	2.94 (5.60)	34 (29–35)	28 (25–31)	3.45 (4.60)	0.51 (-1.07–2.08)
Physical independence (25)	24.5 (22–25)	13 (10–18.75)	8.41 (6.37)	25 (21.5–25)	13 (9–17)	9.39 (5.74)	0.97 (-0.89–2.84)
Pain (35)	33 (31–35)	28 (24–31)	5.44 (4.92)	34 (32–35)	27 (25–29)	6.48 (4.33)	1.04 (-0.38–2.47)
QoR-40 (200)	182 (172–192)	154.5 (136.8–171.8)	26.99 (23.19)	187 (173–192)	149 (135–160.5)	32. 90 (21.51)	5.92 (-0.96–12.79)

The score of each dimension is presented as the median with interquartile range, and the decrement of the score is presented as the mean with standard deviation; * indicates *P* < 0.05

**Table 3:** Result of multivariate linear regression analysis of the decrement of QoR-40

Variable	Coefficient	95% CI of coefficient	t value	VIF
Mode of anesthesia (Remimazolam vs inhalant) *	-7.03	-13.72 – -0.35	-2.08	1.04
Surgical time, min. **	0.11	0.03 – 0.19	2.61	1.34
Sex (Female vs male)	4.43	-5.99 – 14.85	2.61	1.78
Type of surgery (Robotic gynecologic surgery vs laparoscopic cholecystectomy)	3.83	-6.01 – 13.67	0.77	2.17

* indicates *P* < 0.05, ** indicates *P* < 0.01

PONV favored the administration of remimazolam (Table 4). The length of stay in the PACU, hypotension and postoperative pain were comparable in both groups. Postoperative nausea and vomiting were more frequent in patients undergoing robotic gynecologic surgery than in those undergoing laparoscopic cholecystectomy (15 of 97, 15.5% and 2 of 68, 2.9%, respectively). Time to extubation and time to discharge to the PACU were longer in Group R. There were no severe adverse events in the study population.

**Table 4:** Secondary outcomes by group

	Group R	Group D	Relative risk (95% CI)	Mean differences (95% CI)
PONV *	3 (3.7)	14 (16.9)	0.22 (0.06–0.73)	
Pain NRS-11 score ≥ 4	33 (40.2)	42 (50.6)	0.80 (0.57–1.12)	
Administration of rescue analgesics in the PACU	23 (28.0)	29 (34.9)	0.80 (0.51–1.26)	
Intraoperative hypotension	13 (15.9)	17 (20.5)	0.77 (0.40–1.49)	
PACU length of stay, min (IQR)	30 (30–35)	30 (30–30)		
Time to extubation, sec (SD)†	862.0 (292.8)	476.7 (128.6)		385.3 (315.4–455.3)
Time to discharge to the PACU, sec (SD)†	963.5 (306.8)	548.6 (127.0)		414.9 (342.2–487.6)

Presented in numbers with percentages in parentheses; * indicates *P* < 0.05, † indicates *P* < 0.001; *CI* confidence interval, *PACU* postoperative anesthetic care unit, *PONV* postoperative nausea and vomiting, NRS numeric rating scale

## Discussion

In previous studies, TIVA provided a better quality of recovery than balanced anesthesia using inhalant anesthetics and opioids [6, 12, 13]. Remimazolam has some benefits over propofol [14]. Remimazolam has less hemodynamic effects and respiratory suppression than propofol. In addition, patient comfort can be enhanced with less injection pain. Remimazolam has a short context-sensitive half-time, and the drug does not require dose adjustments in subjects with hepatic or renal impairment [15, 16].

In our study, TIVA using remimazolam resulted in a smaller decrement of the global QoR-40 score than conventional balanced anesthesia. The administration of remimazolam can be an option to improve the quality of recovery. There were also trends of less postoperative pain and hemodynamic perturbation, although they were not statistically significant.

Remimazolam induced far less PONV than the inhalant anesthetic. Despite the fact that laparoscopy, cholecystectomy, and gynecologic surgery are considered risk factors for PONV, only 3 patients (3.7%) had PONV following the administration of remimazolam [17]. PONV is often distressing and even possibly dangerous in some patients, and remimazolam could be an appropriate drug for the maintenance of anesthesia for PONV prevention [18, 19].

More time is required for extubation and discharge to the PACU in patients who receive remimazolam. Should recovery be too prolonged after the administration of remimazolam, the rapid reversal of hypnosis can be achieved shortly after the administration of flumazenil. In this kind of situation, flumazenil should be slowly injected to prevent adverse events after administration, such as hypertension or tachycardia [20].

This study was conducted in patients undergoing intraperitoneal laparoscopic surgery, and the course of recovery can be different from other specific types of surgery [11]. The quality of recovery can also be affected by perioperative management, such as an enhanced recovery protocol or premedication [21, 22].

The limitation of this study is that the indication for flumazenil was not defined in advance. Additionally, only the inpatient population was involved in our study. Ambulatory surgical patients constitute a substantial portion of patients in countries such as the United States [23]. A further well-designed study is needed to determine whether remimazolam would be appropriate in outpatient settings.

## Conclusion

Total intravenous anesthesia maintained with remimazolam provides a better quality of recovery than conventional balanced anesthesia in patients undergoing laparoscopic surgery. PONV is far less common with remimazolam administration than inhalant anesthetic administration, but the time of the emergence can be prolonged.

## Data Availability

The datasets used and analyzed during the current study are available from the corresponding author upon reasonable request.

## Abbreviations

ASA: American Society of Anesthesiologist
BIS: Bispectral index
BMI: body mass index
CONSORT: The Consolidated Standards of Reporting Trials
IBW: ideal body weight
PACU: Postoperative anesthetic care unit
PONV: Postoperative nausea and vomiting
TIVA: Total intravenous anesthesia
